# Effect of the Delivery Mode on Pelvic Floor Function and Coping With Birth-Related Pain and Fear: A Prospective Survey Six Months Postpartum

**DOI:** 10.7759/cureus.35065

**Published:** 2023-02-16

**Authors:** Sabine Schütze, Miriam Landenberger, Marlen Heinloth, Juliane Schütze, Sophia Andres, Wolfgang Janni, Miriam Deniz

**Affiliations:** 1 Obstetrics and Gynaecology, University Hospital Ulm, Ulm, DEU; 2 Fundamental Sciences, University of Applied Science, Jena, DEU

**Keywords:** primipara, birth-related fear, birth-related pain, six months postpartum, pelvic floor function

## Abstract

Background and objective

Delivering a baby is one of the most decisive events in a woman`s life and brings along psychological and physical challenges. Therefore, the question arises: which mode of delivery is the best for the woman’s health and her future life? The aim of this study was to evaluate the influence of the delivery mode on pelvic floor function and coping with birth-related pain and fear six months postpartum.

Materials and methods

A total of 200 primiparous women, who delivered during 2018-2019, were included in this prospective case-control study and were asked to fill out the “Pelvic floor questionnaire for pregnant women and women after childbirth” six months after delivery. The women were separated into the following groups: spontaneous vaginal delivery (n = 113), operative vaginal delivery (n = 44), and cesarean section (n = 41). The pelvic floor function as well as coping with birth-related pain and fear six months after delivery was compared.

Results

A significantly higher body mass index was found in the cesarean section group. A significantly worse bladder score was shown in the group with an operative vaginal delivery (p = 0.006). The total score of the questionnaire as well as the anal, prolapse, and sexual subscores showed no difference between the delivery modes. Concerning coping with birth-related pain and fear postpartum, significant differences could be seen between the modes of delivery (pain: p < 0.001; fear: p < 0.001). Women with spontaneous vaginal delivery showed better coping with pain and fear postpartum.

Conclusion

It must be highlighted that women who have had a surgical delivery, including the operative vaginal delivery and cesarean section, stated a lower coping with birth-related pain and fear. This study showed that an operative vaginal delivery has a negative influence on bladder function and the use should be well-indicated. Obstetricians should always be aware of this, as they can contribute to better coping. It is essential to give women the opportunity to talk about the delivery and individual experiences both in pre- and postnatal situations.

## Introduction

Pelvic floor dysfunction is considered by many women as a minor health problem and a natural consequence of childbirth. Historically the well-being of the newborn was the main priority. Nowadays, the role of the mother and the associated potential risk of postpartum physical and psychological problems come into focus. Many postpartum problems originate from pelvic floor dysfunction. Up to 33 % of women suffer from urinary incontinence and 10% report stool incontinence after delivery [1]. Pain after delivery and the coping strategies for birth-related pain and fear have been examined in a few studies but with a low number of participants [2,3]. Khamehchian et al. conducted semi-structured interviews during the first day after spontaneous vaginal delivery (SVD) among 17 primiparous women, demonstrating the role of stress and pain during childbirth [3].

It is known that birth-related pain correlates with postpartum depression, post-traumatic stress [4,5], and worse pelvic floor function years after delivery [6]. All these factors have a huge impact on a woman’s life. Therefore, the question arises of whether the delivery mode has an influence not only on pelvic floor function but also on birth-related pain and fear. The current literature shows conflicting results. Regarding the physical side (in this case, the pelvic floor function), MacLennan et al. stated that cesarean section (CS) is not associated with a significant reduction in long-term pelvic floor morbidity compared to SVD [7]. In contrast, Blomquist et al. showed that CS, compared with SVD, was associated with a significantly lower risk for stress, urinary incontinence, overactive bladder, and pelvic organ prolapse 5-10 years postpartum [8]. On the psychological side, Guittier et al. demonstrated that women with CS reported emotions related to anxiety, fear, disappointment, or feelings of failure [2]. 

The goal of this study was to evaluate the influence of the delivery mode on the physical and psychological well-being of women after childbirth. This included the influence on the pelvic floor function and on coping with birth-related pain and fear six months postpartum of primiparous women.

## Materials and methods

This study was approved by the Ethics Committee of the University of Ulm, Germany (377/16 - FSt/Sta) and registered in the German Clinical Trial Register (DRKS00024725). Informed consent was obtained from all participants. The study was carried out in the University Hospital Ulm, Germany

Study design, participants

Primiparae, who delivered in our hospital during 2018-2019, were asked to take part in this prospective survey study. They were informed about the study by a gynecologist using written and verbal information. Inclusion criteria were the ability to speak German fluently and primiparous women with a singleton pregnancy > 36+6 pregnancy weeks. Exclusion criteria were multiparous women, a premature delivery, multiple pregnancies, and language barriers. For all patients, age, body mass index (BMI), birth weight of the child, and information about epidural anesthesia in case of SVD or operative vaginal delivery (OVD) were collected. The participants received the validated “Pelvic floor questionnaire for pregnant women and women after childbirth” (PFQ) [9] six months postpartum and were asked to answer all questions. For the analysis, the women were divided according to their mode of delivery. There were 11 primary CSs and 30 secondary CSs, both were grouped together to avoid small groups (see Limitations section).

Pelvic floor questionnaire for pregnant women and women after childbirth (PFQ)

The PFQ is a validated questionnaire developed for pregnant women and women after childbirth [9]. This questionnaire contains questions regarding the delivery, anamnestic data, including age, BMI before pregnancy, fetal weight, and 42 questions, which count in the total PFQ score. Besides the total score, the PFQ can be divided into subscores to evaluate the bladder, anal, prolapse, and sexual functions. The subscores can reach values between 0 (no dysfunction) and 10 (dysfunction) and can be calculated separately. These subscores can be added together to get the PFQ total score reaching from 0-40. The higher the value, the worse the pelvic floor function. Even if one answer was missing, the associated subscore and the total score were included in the analysis. Many questions regarding the delivery mode were intended for multiparous women and were therefore not relevant to this study. The following questions were of interest for this study: Did you have pain in your vagina, perineum, or bowel after giving birth? Do you have the feeling that you have dealt with the pain during the delivery and/or afterward? Do you have the feeling that you have dealt with possible fears during childbirth?

Statistical analysis

Data analysis was performed with IBM SPSS Statistics for Windows, Version 26.0 (Released 2019; IBM Corp., Armonk, New York, United States) and Microsoft Excel (V16.3; Microsoft Cooperation, Redmond, Washington, United States). Demographic characteristics were described as medium and range, absolute and relative frequencies for metric, ordinal, and nominal variables respectively. ANOVA and Kruskal-Wallis tests were performed to find mean differences between groups; chi-square tests were used to compare distributions of discrete variables.

## Results

Study population

Out of the 300 women who wanted to take part, 200 completed the questionnaire six months postpartum. Two cases were excluded because they had an OVD attempt and subsequently a CS. Table 1 demonstrates the distribution regarding the mode of delivery and the associated demographic data with a group comparison (Table 1).

**Table 1:** Distribution regarding the mode of delivery and the associated demographic facts with a group comparison.

	Spontaneous vaginal delivery	Operative vaginal delivery	Cesarean section	Group comparison
Number of participants in the group	113	44	41 (11 primary; 30 secondary)	
Percentage of the total participants	57.1%	20.7%	22.2%	
Age	31.49 (SD 4.31)	32 (SD 5.15)	32.83 (SD 4.77)	p = 0.275
Body mass index before delivery	23.69 (SD 4.60)	25.35 (SD 5.07)	25.88 (SD 6.42)	p = 0.032
Fetal birth weight (g)	3298.94 (SD 444.60)	3380.45 (SD 438.03)	3249.88 (SD 508.31)	p = 0.406

Of the participants, 57.1% had an SVD, 20.7% an OVD, and 22.2% a CS. In the group comparison, age and fetal birth weight showed no difference between the groups. The BMI showed a significant difference between the groups (ANOVA p = 0.032 with higher BMI in the CS group)

Pelvic floor function

**Figure 1:**
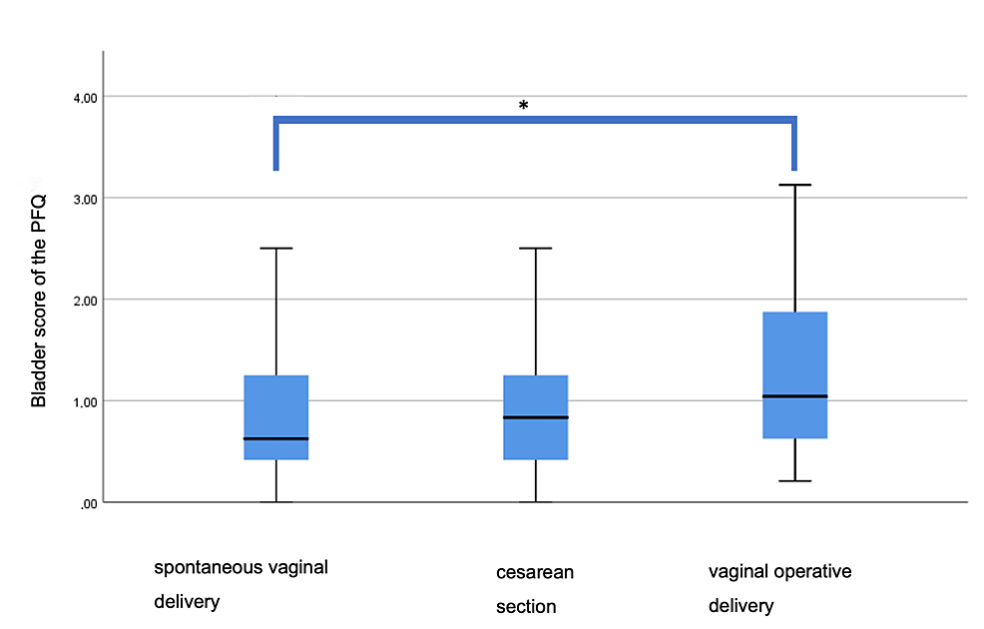
Influence of the delivery mode on the bladder subscore of the PFQ * Significant difference between spontaneous vaginal delivery and the operative vaginal delivery (p = 0.006) PFQ: pelvic floor questionnaire for pregnant women and women after childbirth

Analyzing the influence of the mode of delivery on the pelvic floor function, no difference was found for the total PFQ score between the groups (p = 0.647). There was a significant difference in the bladder subscore (p = 0.008) while all the other subscores showed no significant difference regarding the mode of delivery. A significantly higher bladder score was shown in the OVD group compared to the SVD group (p = 0.006; Figure 1).

Coping with pain and fear

Pain after delivery showed a significant difference between the delivery modes (p < 0.001). In the CS group, only 12.5% of women stated to have pain after delivery compared to 62.5% in the SVD group and 77.3% in the OVD group. In the case of an SVD or an OVD, no difference was found for the stated pain after delivery whether the women had epidural anesthesia or not. 

**Figure 2:**
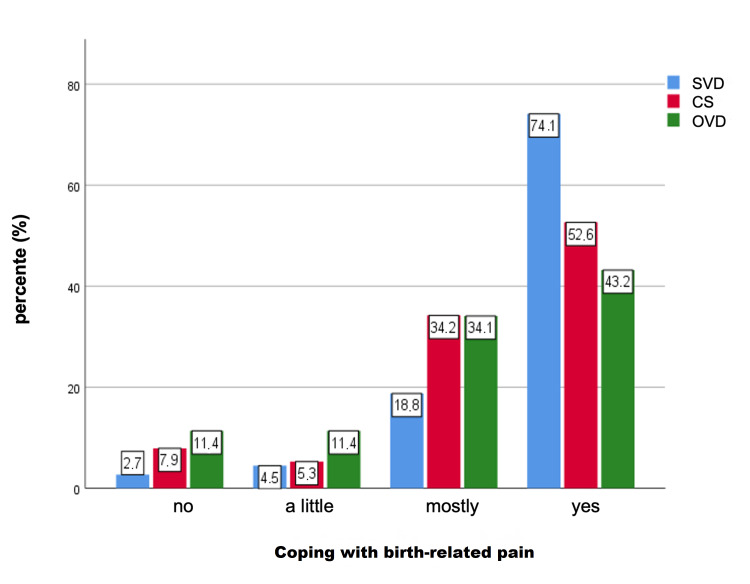
Coping with postpartum birth-related pain between the different modes of delivery (both p < 0.001), with regard to the question: Do you have the feeling that you have dealt with the pain during the delivery and/or afterwards? SVD: spontaneous vaginal delivery; CS: cesarean section; OVD: operative vaginal delivery

Concerning coping with birth-related pain postpartum, 74.1% of participants with SVD stated to have processed the pain completely (comparison: CS: 52.6%; OVD: 43.2%) and in 2.7% to have not processed the pain (comparison: CS 7.9%; OVD 11.4%) (Figure 2).

A significant difference could be shown between the modes of delivery and coping with birth-related pain (p < 0.001). In detail, a worse coping with pain was found for the participants who had an OVD compared to the SVD group (p = 0.001). No significant results were observed between CS/OVD and CS/SVD.

**Figure 3:**
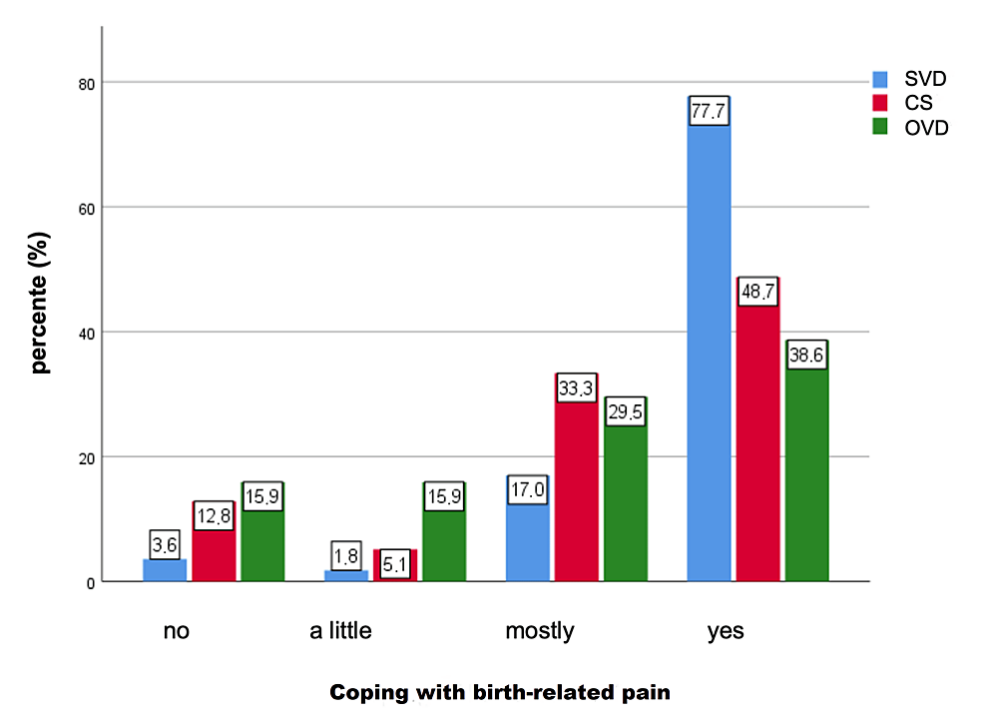
Coping with birth-related fear postpartum in the different modes of delivery (both p < 0.001), with regard to the question: Do you have the feeling that you have dealt with possible fears during childbirth? SVD: spontaneous vaginal delivery; CS: cesarean section; OVD: operative vaginal delivery

Concerning coping with birth-related fear postpartum, 77.7% of the participants with SVD stated that they processed the fear completely (comparison: CS: 48.7%; OVD: 38.6%), and 3.6% stated that they had not processed the fear (comparison: CS 12.8%; OVD 15.9%) (Figure 3).

A significant difference could be seen between the modes of delivery and coping with birth-related fear (p < 0.001). Specifically, significant differences were found between OVD and SVD (p < 0.001) as well as between CS and SVD (p = 0.004).

## Discussion

As one of the most decisive events in a woman’s life, childbirth brings along psychological and physical challenges and therefore there always arises the question of which mode of delivery is the best for the woman’s health and her future life. The aim of this study was to evaluate the influence of the delivery mode on pelvic floor function and on coping with birth-related pain and fear six months postpartum in primiparous women.

In this study, 57.1% of participants had an SVD, 20.7% an OVD, and 22.2% a CS. Compared with the data of Voigt et al., a lower number of CS can be shown in this study with an increased number of OVD. Voigt et al. demonstrated a rise in OVD and CS with increasing maternal age. Women older than 32 years experienced 53.1% VSD, 32.3 % CS, and 11% OVD [10]. Despite age, several studies showed that BMI influences the delivery mode. Dietz et al. examined primiparous women analyzing the number of CS in relation to the maternal BMI, showing an increased rate of CS with rising BMI [11]. The rising number of secondary CS in obese women is due to a known prolonged birth with higher obstetrics risks leading to a more generous indication of CS [12]. This could explain the significantly higher BMI in the CS group in this study.

There is already evidence that the delivery mode influences the physical and psychological outcome after birth. For example, in line with our results, multiple studies showed that OVD is an additional risk factor for pelvic floor function [8], [13-15]. Blomquist et al. compared the influence of the delivery mode on the pelvic floor function 5-10 years postpartum. They showed a significantly higher hazard of anal incontinence and pelvic organ prolapse for women undergoing OVD [8]. However, Crane et al. compared OVD and CS and found no significant impact on pelvic floor function one year after delivery, except for bulge symptoms in the OVD group [16]. In our study, only the bladder function was negatively affected by an OVD. Maybe the rising hazard, as described by Blomquist et al. [8], for anal incontinence and pelvic organ prolapse develops over time and the follow-up period in this study is too short. However, based on the found results it must be emphasized to conduct an OVD only in indicated cases, if the fetal or maternal health needs an intervention.

Regarding pain after delivery, CS is associated with lesser pain than SVD or OVD. This could be due to two factors. On the one hand, the pain after delivery might be not as painful and on the other hand, the exact question was whether there was pain in the genital region and not in the surgical area. The last-mentioned aspect could have falsified the result. In the case of an SVD or an OVD it can be assumed that epidural anesthesia does not protect the development of pain and cannot prevent long-term pain and fear processing.

The best coping with birth-related pain was reported in the SVD group, and the worst in the OVD group. Comparing this with the coping of birth-related fear, similar results were evaluated. These results are surprising, due to the fact that SVD goes along with a high level of pain. It can be assumed, that women prepare themselves cognitively and in prenatal classes for the delivery with the goal of spontaneous delivery. Both OVD and secondary CS are surgical interventions that are not planned and occur in situations where decisions must be made quickly. Therefore, the made plan and wish deviate from reality, and fear appears. Women in these situations reported feelings of helplessness and loss of perceived control in the study of Guittier et al. [2]. Probably many women feel a sense of failure as society paints the picture of an easy spontaneous delivery. The obstetricians should always be aware of this, as they can contribute to better coping with good communication. As recommended in the literature, this study underlines the importance to prepare women during prenatal classes for the eventuality of a CS or an OVD and to offer all women and, if possible, their partners, the opportunity to talk about the experience of childbirth during the postpartum period [2,6]. 

One limitation of this study is the CS group includes both primary and secondary CS. More women should have been included to be able to compare both groups separately. Besides, six months after delivery is a short time and further studies should be conducted to compare a longer follow-up. 

## Conclusions

In summary, OVD has a negative influence on bladder function and its use should be well-indicated. It must be highlighted that the surgical delivery modes, including OVD and CS, have a worse coping with birth-related pain and fear. It is essential to give women the opportunity to talk about the delivery and individual experiences both in pre- and postnatal situations. Besides, obstetricians should always be aware of this, as they can contribute to better coping.
